# Phenotypic and genetic analysis of children with unexplained neurodevelopmental delay and neurodevelopmental comorbidities in a Chinese cohort using trio-based whole-exome sequencing

**DOI:** 10.1186/s13023-024-03214-w

**Published:** 2024-05-19

**Authors:** Ruohao Wu, Xiaojuan Li, Zhe Meng, Pinggan Li, Zhanwen He, Liyang Liang

**Affiliations:** 1 Department of Children’s Neuro-endocrinology, Sun Yat-sen Memorial Hospital, Sun Yat-sen University, Guangzhou, 510120 Guangdong China; 2 Children’s Medical Center, Sun Yat-sen Memorial Hospital, Sun Yat-sen University, Guangzhou Guangdong, 510120 China; 3 Department of Research and Molecular Diagnostics, Sun Yat-sen Memorial Hospital, Sun Yat-sen University, Guangzhou, 510120 Guangdong China

**Keywords:** Trio-based whole-exome sequencing, Neurodevelopmental delay, Neurodevelopmental comorbidities, Autism spectrum disorder, Head circumference abnormality, Diagnostic yield, Autism spectrum disorder-risk genes

## Abstract

**Background:**

Trio-based whole-exome sequencing (trio-WES) enables identification of pathogenic variants, including copy-number variants (CNVs), in children with unexplained neurodevelopmental delay (NDD) and neurodevelopmental comorbidities (NDCs), including autism spectrum disorder (ASD), epilepsy, and attention deficit hyperactivity disorder. Further phenotypic and genetic analysis on trio-WES-tested NDD-NDCs cases may help to identify key phenotypic factors related to higher diagnostic yield of using trio-WES and novel risk genes associated with NDCs in clinical settings.

**Methods:**

In this study, we retrospectively performed phenotypic analysis on 163 trio-WES-tested NDD-NDCs children to determine the phenotypic differences between genetically diagnosed and non-genetically diagnosed groups. Additionally, we conducted genetic analysis of ASD genes with the help of Simons Foundation for Autism Research Institute (SFARI) Gene database to identify novel possible ASD-risk genes underlying genetic NDD conditions.

**Results:**

Among these 163 patients, pathogenic variants were identified in 82 cases (82/163, 50.3%), including 20 cases with CNVs. By comparing phenotypic variables between genetically diagnosed group (82 cases) and non-genetically diagnosed group (81 cases) with multivariate binary logistic regression analysis, we revealed that NDD-NDCs cases presenting with severe-profound NDD [53/82 *vs* 17/81, adjusted-OR (95%CI): 4.865 (2.213 – 10.694), adjusted-*P* < 0.001] or having multiple NDCs [26/82 *vs* 8/81, adjusted-OR (95%CI): 3.731 (1.399 – 9.950), adjusted-*P* = 0.009] or accompanying ASD [64/82 *vs* 35/81, adjusted-OR (95%CI): 3.256 (1.479 – 7.168), adjusted-*P* = 0.003] and head circumference abnormality [33/82 *vs* 11/81, adjusted-OR (95%CI): 2.788 (1.148 – 6.774), adjusted-*P* = 0.024] were more likely to have a genetic diagnosis using trio-WES. Moreover, 37 genes with monogenetic variants were identified in 48 patients genetically diagnosed with NDD-ASD, and 15 dosage-sensitive genes were identified in 16 individuals with NDD-ASD carrying CNVs. Most of those genes had been proven to be ASD-related genes. However, some of them (9 genes) were not proven sufficiently to correlate with ASD. By literature review and constructing protein-protein interaction networks among these 9 candidate ASD-risk genes and 102 established ASD genes obtained from the SFARI Gene database, we identified *CUL4B*, *KCNH1*, and *PLA2G6* as novel possible ASD-risk genes underlying genetic NDD conditions.

**Conclusions:**

Trio-WES testing is recommended for patients with unexplained NDD-NDCs that have severe-profound NDD or multiple NDCs, particularly those with accompanying ASD and head circumference abnormality, as these independent factors may increase the likelihood of genetic diagnosis using trio-WES. Moreover, NDD patients with pathogenic variants in *CUL4B*, *KCNH1* and *PLA2G6* should be aware of potential risks of developing ASD during their disease courses.

**Supplementary Information:**

The online version contains supplementary material available at 10.1186/s13023-024-03214-w.

## Introduction

Neurodevelopmental delay (NDD) is a group of common neurological diseases with high clinical heterogeneity during childhood [1] and affects approximately 1%–3% of children worldwide, resulting in an average lifetime cost of $1 million to support the affected child [2]. Global developmental delay/intellectual disability (GDD/ID) is the most common and representative manifestation in NDD [3]. GDD is defined as a pathological delay or failure to achieve milestones in a minimum of two of the five developmental domains: gross motor skills, fine motor skills, speech/language skills, social cognitive skills, and social/emotional skills. By the age of five, most patients with GDD will present with ID, which is characterized by limitations in social adaptability and an intelligence quotient (IQ) score <70 [3].

GDD/ID is a highly complex disorder, development and progression of which can be influenced by various genetic and environmental factors. GDD/ID pathogenesis is closely associated with genetic alterations, and genetic causes are considered to have an essential role in GDD/ID. For example, single nucleotide variants (SNVs), copy-number variants (CNVs), and aneuploidies lead 30%–50% of cases of GDD/ID [4], and fragile X, Rett, and Down syndromes are the three most common types of syndromic GDD/ID in the world [5]. Due to the rapid development of next-generation sequencing technologies, particularly the popularization of trio-based (parental-offspring model) whole-exome sequencing (trio-WES) technology - a comprehensive genetic analysis enabling the detection of SNVs and CNVs, genetic causes are being identified more frequently than before in many unexplained or idiopathic GDD/ID cases [6]. To date, over 1300 causative genes and 1100 candidate genes related to GDD/ID pathogenesis have been identified, and the number continues to grow annually [7].

Other common neurodevelopmental disorders, particularly autism spectrum disorder (ASD), epilepsy (EP), and attention deficit hyperactivity disorder (ADHD), can also impact brain development and affect various aspects of daily functioning in childhood [8]. ASD is a genetically and clinically heterogeneous disorder, characterized by social communication dysfunctions and repetitive, stereotypic patterns of movement and behavior [9]. EP is among the most common neurological conditions in children, and is characterized by repeated seizures and unexpected disturbances of brain electrical activity. The pathogenesis of EP, particularly epileptic encephalopathy, is thought to have a genetic basis [10]. ADHD is another common childhood-onset behavioral disorder with high genetic heterogeneity, characterized by persistent inattention and/or hyperactive-impulsive behavior, resulting in impaired social functioning [11]. ASD, EP, and ADHD are the three most frequent neurodevelopmental comorbidities among patients with NDD (NDD-NDCs) with a known genetic cause [12]. For example, the prevalence of GDD/ID in patients with genetic EP is higher than that in patients with non-genetic EP [13]. Additionally, many children with genetic ASD presented with GDD/ID phenotypes [14]. Moreover, Kuntsi et al. found that almost all children with genetic ADHD presented with GDD/ID phenotype of varying severity [15]. Further phenotypic analysis with trio-WES data of unexplained NDD-NDCs patients may help to identify key phenotypic features related to higher diagnostic yield of using trio-WES in NDD-NDCs conditions.

On the other hand, a recent large WES study of over 11000 ASD individuals with a total of 35000 samples published in *Cell* [16] showed that most identified ASD-risk genes have essential roles in neuronal communication, gene expression regulation, and metabolism. These functional pathways are also involved in the genetic etiology of NDD [17]. Similar to genetic NDD, the genetic spectrum of ASD is also constantly expanding; however, it is difficult to identify novel ASD-risk genes in single-center studies, due to limited numbers of cases of isolated ASD. As previously described, ASD-risk genes have increasingly been found to be involved in the pathogenesis of NDD [8], we hypothesized that the likelihood of identifying novel possible ASD-risk genes would increase if we focused our efforts on subjects with genetic NDD and comorbid ASD (NDD-ASD).

This single-center study summarized clinical features of 163 unexplained NDD-NDCs patients with trio-WES testing. By comparing the phenotypic difference between genetically diagnosed and non-genetically diagnosed NDD-NDCs patients, we revealed that the presence of severe NDD, multiple NDCs and the accompanying ASD or head circumference abnormality in unexplained NDD-NDCs patients leads to a higher trio-WES diagnostic yield. Moreover, by categorizing ASD-risk genes in individuals with genetic NDD-ASD and constructing protein-protein interaction (PPI) networks between candidate ASD-risk genes and established ASD genes, we identified novel possible ASD-risk genes underlying genetic NDD conditions, providing new insights into genetic alterations and molecular mechanisms potentially shared between NDD and ASD.

## Methods

### Criteria for participant enrollment and variant capture strategy

The medical records of independent children with unexplained NDD (GDD/ID) and one or more NDCs, including ASD, EP, and ADHD, who had undergone trio-WES and were admitted to the Children’s Medical Center of Sun Yat-sen Memorial Hospital, Sun Yat-sen University from October 2018 to December 2022, were reviewed. Clinical and laboratory baseline data were collected from records for patients who met the following eligibility criteria: (a) clear and complete baseline clinical and laboratory data, including clinical manifestations (severity of GDD/ID, history of ASD, EP and ADHD, comorbid organ anomalies, birth condition and family history), and findings of electroencephalogram (EEG), cranial magnetic resonance imaging (MRI), auditory brainstem response/visual evoked potentials, echocardiography/abdominal ultrasonography; (b) clear and complete trio-WES data and had undergone systematic clinical examinations to exclude common non-genetic causes, such as cerebral ischemia, hypoxia and injury; and (c) negative results of routine genetic screening tests, including G-band karyotyping and triplet repeat primed PCR followed by fragment analysis of *FMR1* gene CGG repeats detection. Finally, 163 eligible children with unexplained NDD-NDCs were enrolled in this retrospective research.

Clinical diagnostic criteria for GDD/ID were based on the Diagnostic and Statistical Manual of Mental Disorders, 5^th^ Edition (DSM-V) guidelines for GDD/ID [3]. Specifically, developmental quotient (DQ) was used to evaluate the developmental scale in five domains, and children with DQ scores <75 in at least two domains were diagnosed with GDD [18]. For individuals older than five years, we used the Wechsler Intelligence Scale for Children to assess IQ scores of subjects; children with IQ scores <70 were diagnosed with ID [19]. Clinical diagnoses of ASD and ADHD were made following the DSM-V diagnostic criteria for ASD and ADHD [3], along with additional clinical assessments, such as the Autism Behavior Checklist [20], Childhood Autism Rating Scale [21], or Modified Checklist for Autism in Toddlers [22]. The International League Against Epilepsy criteria were used to diagnose EP, epileptic syndromes, and epileptic encephalopathy [23]. Moreover, the International Classification of Diseases version-10 classification criteria were used to assess the severity of NDD [24]. Specifically, a patient with GDD and at least two DQ domains with scores <35 points was considered to have severe-profound GDD, otherwise, he or she was considered to have mild-moderate GDD. In addition, a patient with ID and an IQ score ranging from 40–70 points was regarded to have mild-moderate ID, while those with IQ score <40 points were diagnosed with severe-profound ID. Abnormalities in head circumference, including microcephaly and macrocephaly, and facial anomalies, as well as various other types of organ anomalies, were defined based on the Human Phenotype Ontology guidelines [25].

The variant capture strategy for trio-WES can be briefly summarized as follows: DNA samples were extracted from peripheral blood samples (approximately 2 ml) from each family member using a QIAamp DNA Mini Kit (QIAGEN, Hilden, Germany). DNA libraries were generated using an Illumina TruSeq Exome Kit, according to the manufacturer’s protocol (Illumina, San Diego, CA, USA) and then sequenced on Illumina Novaseq 6000 platforms (Illumina, San Diego, CA, USA). Approximately 10 GB of data were obtained per individual, and quality control assessment performed using the FastQC toolkit (Babraham Bioinformatics, London, UK), with >97.5% of targeted regions sequenced to a depth of 20× (mean depth of coverage, approximately100×). Next, sequences were aligned to the human GrCh37/hg19 reference sequence using the Burrows-Wheeler Aligner to output Binary Alignment Map-format (BAM) files. BAM files were processed using Picard software (version: 2.18.2), along with realignment of indel regions and base quality recalibration. Genome Analysis Toolkit HaplotypeCaller (version: 4.0.4) was used for variant calling to create variant call format (VCF) files. Finally, VCF files were annotated using ANNOVAR software (version: 2019/10/24). Variants were filtered based on quality/coverage depth (≥ 10) and minor allele frequency <0.05%, according to the Genome Aggregation Database (gnomAD). Then, variants were filtered according to the proband’s phenotype, inheritance pattern, clinical significance, and reported clinically relevant variants. Candidate CNVs and SNVs detected by trio-WES were confirmed by chromosomal microarray analysis (CMA) and Sanger sequencing in probands and their parents, respectively, if necessary. The primer sequences used for Sanger sequencing in the current research for all detected NDD-NDCs-related pathogenic SNVs are presented in Supplementary File 1.

### Criteria for variant pathogenicity rating and ethical compliance

For candidate SNVs, the pathogenicity rating was compiled to the 2015 ACMG guidelines and detected SNVs were accordingly classified as "benign/uncertain significance SNV", "likely pathogenic SNV" and "pathogenic SNV" [26]. SNV allele frequency was determined by referring to gnomAD [27] and our in-house SNV population frequency database. Local versions of REVEL [28], Polyphen-2 [29], SIFT [30], Mutation Taster [31], and PROVEAN [32] were used for *in silico* prediction of the pathogenicity of detected missense/nonsense/frameshift/deletion variants. In addition, Human Splicing Finder software [33] and CADD [34] were applied to predict the pathogenicity of candidate splice variants. PubMed, Human Genomic Mutation Database [35, 36], and ClinVar [37] were consulted to determine whether candidate variants had been reported previously. Online Mendelian Inheritance in Man (OMIM) and GeneReviews (https://www.ncbi.nlm.nih.gov/books/NBK1116) were searched for genotype and phenotype profiles related to detected SNVs.

The pathogenicity of candidate CNVs was predicted based on the 2019 guidelines of the ACMG for the interpretation of postnatal CNVs [38] and using the detailed procedure previously reported [39]. All candidate CNVs in the current study were manually interpreted and classified as “benign/uncertain significance CNV” and “likely pathogenic/pathogenic CNV” by experienced clinicians and clinical geneticists, following the ACMG guidelines.

Final genetic diagnoses were made by the multi-disciplinary team, consisting of specialized pediatric neurologists, child psychologists, and clinical geneticists from Sun Yat-sen Memorial Hospital, Sun Yat-sen University. The design of this retrospective study was in accordance with the Declaration of Helsinki and this study was approved by the Ethics Committee of the Sun Yat-sen Memorial Hospital (Approval Number: SYSKY-2023-336-01). Written informed consent was obtained from the parents or guardians of all 163 enrolled subjects.

### Statistical methods for phenotypic and genotype-phenotype analyses

Traditional statistical analyses were applied to explore the significant phenotypic and genotype-phenotype characteristics of patients with genetic NDD-NDCs and phenotypic differences between patients with genetically diagnosed and non-genetically diagnosed NDD-NDCs following trio-WES. All statistical analyses were performed using SPSS software (version 22.0) (IBM Inc., NY, USA). Specifically, Chi-squared test was used to compare qualitative phenotypic variables, such as percentages of ASD, EP, and head circumference abnormality, between mild-moderate and severe-profound GDD/ID groups; and qualitative phenotypic variables, such as NDD severity, and numbers and types of NDCs, between the genetically diagnosed and non-genetically diagnosed groups. An independent Student’s *t*-test was used to compare quantitative variables, such as age at trio-WES testing, between the genetically diagnosed and non-genetically diagnosed groups. Univariate and multivariate binary logistic regression analysis were then used to analyze odds radio (OR) with 95% confidence interval (95%CI) of phenotypic variables between the genetically diagnosed and non-genetically diagnosed groups and further confirm independent effects from identified phenotypic variables on diagnosis made based on trio-WES. Likewise, the Chi-squared test was used to compare qualitative genotypic variables, including SNV/CNV percentages, *de novo*/non-*de novo*, and truncation/non-truncation variants, between mild-moderate and severe-profound GDD/ID groups, similar to the approach proposed by Liu et al. in their recent cohort study of genetic infantile spasms [40]. *P* < 0.05 was considered statistically significant.

### Screening possible mutated genes related to ASD

Mutated genes potentially related to ASD phenotype in our genetically diagnosed NDD-ASD patients were screened using the Simons Foundation for Autism Research Institute (SFARI) Gene database, combined with PPI network analysis and literature review using the OMIM database (https://omim.org). The main principle of this method was derived from a recent genetic NDD-ASD cohort study reported by Chen et al.; by applying this method, they identified a novel possible ASD-risk gene from their cohort of patients with genetic NDD-ASD [8]. SFARI Gene database [41] is a powerful public database that can grade SNV genes and CNVs related to genetic ASD phenotype with different correlation levels by referring to the number of reported cases or molecular functional experimental findings of mutated genes/CNVs reported in the publications. We use the SFARI Gene database as follows:

First, all detected SNVs and CNVs, along with dosage-sensitive genes within CNVs, were grouped into four classes, according to the strength of their association with autistic phenotypes, based on the SFARI Gene database rating criteria. Mutated genes containing SNVs were classified as follows: (1) Class 1: mutated genes clearly associated with ASD etiology and presented as category 1 (high-confidence) and category S (syndromic ASD) in the SFARI Gene database; (2) Class 2: mutated genes with two or more reports of ASD-related cases with *de novo* likely gene-disrupting mutations, or ASD susceptibility supported by a genome-wide association study, or a molecular functional effect associated with ASD etiology, and were presented as category 2 (strong evidence) in the SFARI Gene database; (3) Class 3: mutated genes with only one ASD-related case report with *de novo* likely gene-disrupting mutations or inherited variants that had no rigorous statistical comparisons and were categorized into category 3 (suggestive evidence) in the SFARI Gene database; and (4) Class 4: mutated genes were not included in the SFARI Gene database or found in the SFARI Gene database but did not meet the criteria for categories S, 1, 2, 3. Detected CNVs, including dosage-sensitive genes, were classified as follows: (1) Recorded CNVs with ASD-related genes: Both CNVs and their included dosage-sensitive genes (categories S, 1, 2, and 3) were recorded and clearly implicated in ASD susceptibility in the SFARI Gene database; (2) Recorded CNVs without ASD-related genes: CNVs were recorded in the SFARI Gene database, but their included dosage-sensitive genes did not meet the criteria for categories S, 1, 2, 3, or were not included in the SFARI Gene database; (3) Unrecorded CNVs with ASD-related genes: Only the included dosage-sensitive genes met the criteria for categories S, 1, 2, 3 in the SFARI Gene database, while corresponding CNVs were not recorded in the SFARI Gene database; and (4) Unrecorded CNVs without ASD-related genes: neither the CNVs nor their included dosage-sensitive genes were present in the SFARI Gene database.

Second, only genes from Class 4 and dosage-sensitive genes in the “Recorded CNVs without ASD-related genes” group were considered as candidate ASD-risk genes, with low strength associations with ASD phenotypes based on previous evidence. To provide more evidence and identify novel possible ASD-risk genes from among candidate ASD-risk genes, PPI network analysis was conducted using interaction data from STRING [42] to further determine whether candidate genes with weak ASD correlations directly interact with any of 102 established ASD-related genes obtained from the SFARI Gene database (Supplementary File 2) at the protein levels. Candidate genes that had strong and multiple interactions with established ASD-related genes were considered novel possible ASD-risk genes in the current research. Genes that had no interaction with ASD-related genes, but were associated with ASD phenotype in at least four cases in our cohort and other cohorts were also considered to be novel possible ASD-risk genes.

## Results

### Diagnosis categories and phenotypic characteristics of the cohort genetically diagnosed with NDD-NDCs

**Fig. 1:**
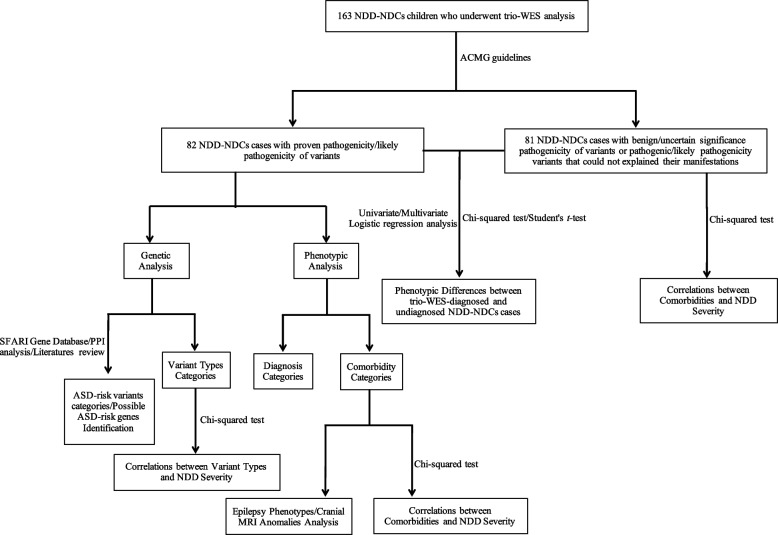
Flowchart for this study. NDD-NDCs, neurodevelopmental delay with neurodevelopmental comorbidities; trio-WES, trio-based whole-exome sequencing; MRI, magnetic resonance imaging; ACMG, American College of Medical Genetics; NDD, neurodevelopmental delay; PPI, protein-protein interaction; ASD, autism spectrum disorder

A flowchart of this study is presented as Fig. 1. After rating the pathogenicity of variants identified by trio-WES in the 163 enrolled children with NDD-NDCs according to the ACMG guidelines, 82 subjects were confirmed to carry pathogenic or likely pathogenic variants related their clinical manifestations, while genetic diagnosis was not obtained by trio-WES for 79 subjects, due to the benign or uncertain significance of identified variants. Two individuals carried pathogenic or likely pathogenic variants, but the variants could not explain their NDD-NDCs manifestations. One case was homozygous for the *HBA2* variant [NM_000517.6: c.377T>C(p.Leu126Pro)], which was inherited from both parents, and was diagnosed with alpha thalassemia; the other patient carried a *de novo GNAS* variant [NM_000516.7: c.139G>A(p.Gly47Ser)] and was diagnosed with pseudohypoparathyroidism Ia.

Finally, 81 and 82 unrelated subjects were included in the non-genetically diagnosed and genetically diagnosed groups, respectively. The global diagnostic yield of trio-WES in our study was 50.3%. Detailed information about the clinical phenotypes and genotypes of the 82 cases with genetically diagnosed NDD-NDCs are summarized in Supplementary Files 3 and 4, respectively. The male:female ratio of the 82 genetically diagnosed cases was 1.83:1, and their mean ± standard error age at genetic diagnosis was 4.50 ± 0.35 years (median, 3 years-old).

As shown in Table 1, of the 82 genetically diagnosed children, 62 (75.6%) patients were diagnosed with SNV-mediated syndromes and 20 (24.4%) with CNV-mediated syndromes. Among SNV syndromes, Rett syndrome (4/62, 6.5%) was the most frequent, followed by autosomal dominant mental retardation type 35 (3/62, 4.8%), Okur-Chung neurodevelopment syndrome (OCNS) (3/62, 4.8%), Coffin-Lowry syndrome (2/62, 3.2%), Floating-Harbor syndrome (2/62, 3.2%), Sotos syndrome (2/62, 3.2%), Wiedemann-Steiner syndrome (2/62, 3.2%), X-linked mental retardation type 1 (2/62, 3.2%), neurodegeneration with brain iron accumulation type 2B (NBIA2B) (2/62, 3.2%), and *SCN1A*-related epileptic encephalopathy (2/62, 3.2%). Of CNV syndromes, Chromosome 15q11-q13 microdeletion syndrome (*UBE3A* involved) (3/20, 15.0%) was the most frequent, followed by Chromosome 1q21.1 microdeletion syndrome (*GJA5* involved) (2/20, 10.0%).

**Table 1:** General clinical features and phenotypes in 82 genetic NDD-NDCs children diagnosed by trio-WES

**Characteristics**	**Number (%)**
SNV syndromes:	62/82 (75.6%)
Rett syndrome	4/62 (6.5%)
Autosomal dominant mental retardation type 35	3/62 (4.8%)
Okur-Chung neurodevelopment syndrome	3/62 (4.8%)
Coffin-Lowry syndrome	2/62 (3.2%)
Floating-Harbor syndrome	2/62 (3.2%)
Sotos syndrome	2/62 (3.2%)
Wiedemann-Steiner syndrome	2/62 (3.2%)
X-linked mental retardation type 1	2/62 (3.2%)
Neurodegeneration with brain iron accumulation type 2B	2/62 (3.2%)
*SCN1A*-related epileptic encephalopathy	2/62 (3.2%)
CNV syndromes:	20/82 (24.4%)
Chromosome 15q11-q13 microdeletion syndrome (*UBE3A* involved)	3/20 (15.0%)
Chromosome 1q21.1 microdeletion syndrome (*GJA5* involved)	2/20 (10.0%)
GDD/ID severity:	
Mild-moderate	29/82 (35.4%)
Severe-profound	53/82 (64.6%)
NDCs:	
ASD	64/82 (78.0%)
EP	26/82 (31.7%)
ADHD	22/82 (26.8%)
Organ anomalies/dysfunctions	
Dysmorphic facial feature	59/82 (72.0%)
Abnormal cranial MRI	44/82 (53.7%)
Head circumference abnormality (microcephaly/macrocephaly)	33/82 (40.2%)
Abnormal EGG	23/82 (28.0%)
Respiratory/immune dysfunctions	19/82 (23.2%)
Oral cavity&Gastrointestinal/abdominal disorders	17/82 (20.7%)
SS	15/82 (18.3%)
CHDs	15/82 (18.3%)
Ear anomalies/hearing loss	12/82 (14.6%)
Reproductive/endocrine abnormalities	12/82 (14.6%)
Skin/hair changes	11/82 (13.4%)
Skeletal/muscle anomalies	6/82 (7.3%)
Ocular/visual dysfunctions	6/82 (7.3%)
Renal anomalies	4/82 (4.9%)

*NDD-NDCs* Neurodevemental delay and neurodevelopmental comorbidities, *GDD/ID* Global developmental delay/intellectual disability, *SNV* Single nucleotide variant, *CNV* Copy-number variant, *NDCs* Neurodevelopmental comorbidities, *ASD* Autism spectrum disorder, *EP* Epilepsy, *ADHD* attention deficit hyperactivity disorder, *CHDs* Congenital heart defects, *EGG* Electroencephalogram, *MRI* magnetic resonance imaging, *SS* Short stature, *trio-WES* Trio-based whole-exome sequencing

Among the 82 patients with genetically diagnosed NDD-NDCs (Table 1), 53 (64.6%) had severe-profound GDD/ID, and the remainder (29/82, 35.4%) had mild-moderate GDD/ID. Most patients (64/82, 78.0%) had comorbid ASD, while 26 (31.7%) and 22 (26.8%) had comorbid EP and ADHD, respectively. Organ anomaly/dysfunction comorbidities included dysmorphic facial features (59/82, 72.0%), abnormal cranial MRI (44/82, 53.7%), head circumference abnormality (33/82, 40.2%) [microcephaly (24/82, 29.3%), macrocephaly (9/82, 10.9%)], abnormal EEG (23/82, 28.0%), respiratory/immune dysfunction (19/82, 23.2%), oral cavity and gastrointestinal/abdominal disorders (17/82, 20.7%), short stature (15/82, 18.3%), congenital heart defects (CHDs) (15/82, 18.3%), ear anomalies/hearing loss (12/82, 14.6%), reproductive/endocrine abnormalities (12/82, 14.6%), skin/hair changes (11/82, 13.4%), skeletal/muscle abnormalities (6/82, 7.3%), ocular/visual dysfunctions (6/82, 7.3%), and renal anomalies (4/82, 4.9%).

Genetically diagnosed cases were divided into two groups based on the severity of NDD: mild to moderate and severe to profound GDD/ID groups. As shown in Table 2, patients with severe-profound GDD/ID appeared to more often have comorbid ASD (45/53 *vs* 19/29, *P* = 0.043) than those with mild-moderate GDD/ID. Comorbid EP (13/53 *vs* 13/29, *P* = 0.059), ADHD (14/53 *vs* 8/29, *P* = 0.909), and other comorbidities, such as facial anomalies, hearing loss, CHDs, skin/hair changes and skeletal abnormalities, did not differ significantly between the mild-moderate and severe-profound GDD/ID groups (all *P* > 0.05). More head circumference abnormalities, including microcephaly and macrocephaly, were observed in cases with severe-profound GDD/ID than in those with mild-moderate GDD/ID (26/53 *vs* 7/29, *P* = 0.028). An EP/seizure phenotype was found in 26 individuals (26/82, 31.7%), and detailed information about the EP/seizure phenotype and related genotype of these 26 cases are summarized in Supplementary Files 5. Moreover, cranial MRI abnormalities were detected in 44 individuals with genetically diagnosed NDD-NDCs (44/82, 53.5%), and detailed information about the cranial MRI abnormalities and related genotype of these 44 patients are summarized in Supplementary Files 6

**Table 2:** Comparison of neurodevelopmental comorbidities and organ anomalies/dysfunctions between mild-moderate and severe-profound GDD/ID in 82 genetic NDD-NDCs children diagnosed by trio-WES

	**Mild-moderate (*N*=29)**	**Severe-profound (*N*=53)**	***P* value**
ASD	19, 65.5%	45, 84.9%	0.043*
EP	13, 44.8%	13, 24.5%	0.059
ADHD	8, 27.6%	14, 26.4%	0.909
Dysmorphic facial features	18, 62.1%	41, 77.4%	0.141
Abnormal cranial MRI	14, 48.3%	30, 56.6%	0.470
Head circumference abnormality (microcephaly/macrocephaly)	7, 24.1%	26, 49.1%	0.028*
Abnormal EGG	11, 20.7%	12, 22.6%	0.141
Respiratory/immune dysfunctions	7, 24.1%	12, 22.6%	0.878
Oral cavity&Gastrointestinal/abdominal disorders	8, 27.6%	9, 17.0%	0.257
SS	5, 17.2%	10, 18.9%	0.855
CHDs	3, 10.3%	12, 22.6%	0.168
Ear anomalies/hearing loss	2, 6.9%	10, 18.9%	0.143
Reproductive/endocrine abnormalities	4, 13.8%	8, 15.1%	0.873
Skin/hair changes	6, 20.7%	5, 9.4%	0.153
Skeletal/muscle anomalies	3, 10.3%	3, 5.7%	0.436
Ocular/visual dysfunctions	1, 3.4%	5, 9.4%	0.320
Renal anomalies	1, 3.4%	3, 5.7%	0.657

*NDD-NDCs* Neurodevemental delay and neurodevelopmental comorbidities, *GDD/ID* Global developmental delay/intellectual disability, *ASD* Autism spectrum disorder, *EP* Epilepsy, *ADHD* Attention deficit hyperactivity disorder, *SS* Short stature, *CHDs* Congenital heart defects, *EGG* electroencephalogram, *MRI* Magnetic resonance imaging, *trio-WES* Trio-based whole-exome sequencing.* *P* <0.05

### Phenotypic differences between the genetically diagnosed and non-genetically diagnosed NDD-NDCs cohorts

**Fig. 2:**
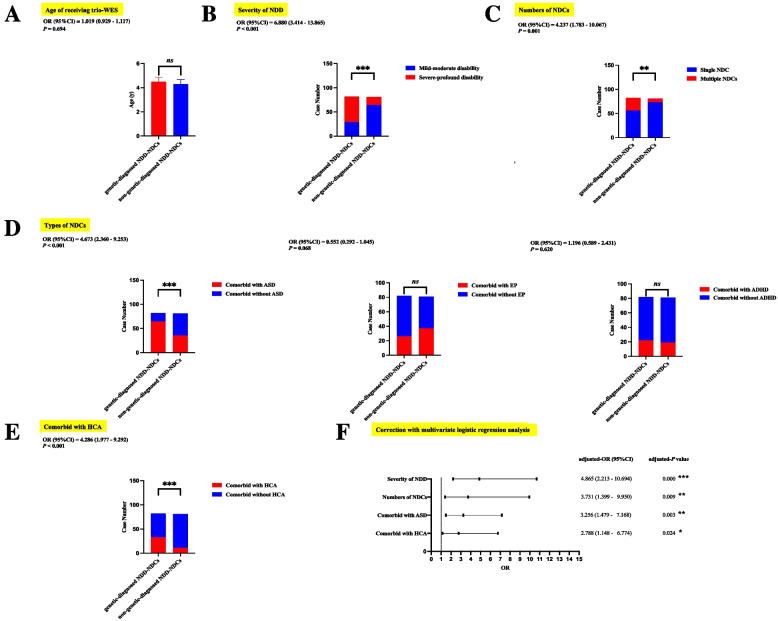
Phenotypic differences between genetically diagnosed and non-genetically diagnosed cases in our cohort of 163 children with unexplained NDD-NDCs analyzed by trio-WES. Differences in (**A**) trio-WES testing age, **B** NDD severity, **C** NDC number (single NDC, NDD children with one type of NDC; multiple NDCs, NDD children with at least two types of NDC), **D** NDC types (including ASD, ADHD, and EP) and (**E**) HCA comorbidity using Student’s *t*-test/Chi-squared test with univariate logistic regression analysis. **F** Forest plots showing independent effects from NDD severity, NDC number, ASD and HCA comorbidities on diagnosis made by trio-WES in our cohort after adjusting collinearity effects and potential confounding factors by using multivariate logistic regression analysis. NDD-NDCs, neurodevelopmental delay with neurodevelopmental comorbidities; trio-WES, trio-based whole-exome sequencing; NDD, neurodevelopmental delay; NDCs, neurodevelopmental comorbidities; ASD, autism spectrum disorder; ADHD, attention deficit hyperactivity disorder; EP, epilepsy; HCA, head circumference abnormality; OR, odds ratio; 95%CI, 95% confidence interval. * *P* <0.05; ** *P* <0.01; *** *P* <0.001

Given our findings that a severe NDD phenotype was more likely to be associated with head circumference abnormality (mainly microcephaly) and ASD in the genetically diagnosed NDD-NDCs cohort, we next explored whether those phenotypic correlations also occurred in the non-genetically diagnosed NDD-NDCs cohort (81 cases). As demonstrated in Supplementary File 7 and Table 3, cases with severe-profound GDD/ID in the non-genetically diagnosed NDD-NDCs group were also more likely to have microcephaly than those with mild-moderate GDD/ID (5/17 *vs* 6/64, *P* = 0.032); however, the positive correlation between NDD severity and ASD comorbidity frequency was absent in the non-genetically diagnosed NDD-NDCs group (7/17 *vs* 28/64, *P* = 0.849). In addition, there was no significant difference in age at trio-WES analysis between the genetically diagnosed and non-genetically diagnosed NDD-NDCs groups (4.50 ± 0.35 *vs* 4.29 ± 0.39, *P* = 0.694) (Fig. 2A). Importantly, by adopting Chi-squared test with univariate logistic regression analysis, we found NDD-NDCs cases presenting with severe-profound GDD/ID [53/82 *vs* 17/81, OR (95%CI): 6.880 (3.414 – 13.865), *P* < 0.001] or having multiple NDCs [26/82 *vs* 8/81, OR (95%CI): 4.237 (1.783 – 10.067), *P* = 0.001] were more likely to have a positive trio-WES result (Fig. 2B,C). Moreover, the presence of ASD comorbidity [64/82 *vs* 35/81, OR (95%CI): 4.673 (2.360 – 9.253), *P* < 0.001] (Fig. 2D) and head circumference abnormality [33/82 *vs* 11/81, OR (95%CI): 4.286 (1.977 – .9.292), *P* < 0.001] (Fig. 2E) in our patients also increased the odds of having a genetic diagnosis made by trio-WES. However, as the reliabilities of these multiple comparisons only using univariate logistic regression analysis were vulnerable to potential confounders and collinearity effects, multivariate binary logistic regression analysis was then performed to confirm independent effects from these four significant phenotypic factors on diagnosis made by trio-WES. After correction with multivariate logistic regression analysis, we further determined that NDD severity, NDC number, the presence of ASD comorbidity and head circumference abnormality were all independently associated with a positive trio-WES result with adjusted-OR (95%CI) of 4.865 (2.213 – 10.694, adjusted-*P* < 0.001), 3.731 (1.399 – 9.950, adjusted-*P* = 0.009), 3.256 (1.479 – 7.168, adjusted-*P* = 0.003) and 2.788 (1.148 – 6.774, adjusted-*P* = 0.024), respectively (Fig. 2F).

**Table 3:** Comparison of neurodevelopmental comorbidities and head circumference abnormality between mild-moderate and severe-profound GDD/ID in 81 non-genetically diagnosed NDD-NDCs children

	**Mild-moderate (*N*=64)**	**Severe-profound (*N*=17)**	***P* value**
ASD	28, 43.8%	7, 41.2%	0.849
EP	29, 45.3%	8, 47.1%	0.898
ADHD	16, 25.0%	3, 17.6%	0.525
Head circumference abnormality (microcephaly)	6, 9.4%	5, 29.4%	0.032*

*NDD-NDCs* Neurodevemental delay and neurodevelopmental comorbidities, *GDD/ID* Global developmental delays/intellectual disability, *ASD* Autism spectrum disorder, *EP* Epilepsy, *ADHD* attention deficit hyperactivity disorder, *trio-WES* Trio-based whole-exome sequencing.* *P* <0.05

### Genotypic features of the genetically diagnosed NDD-NDCs cohort

**Fig. 3:**
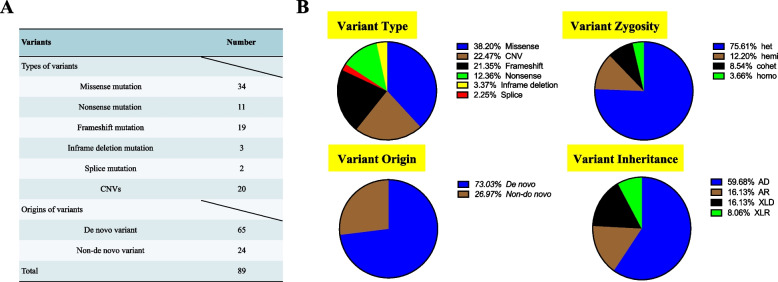
Details of 89 variants identified in individuals with NDD-NDCs by trio-WES. **A** Number of detected variants in different types and origins. **B** Proportions of variant type, zygosity, origins and inheritance pattern among detected variants. NDD-NDCs, neurodevelopmental delay with neurodevelopmental comorbidities; trio-WES, trio-based whole-exome sequencing; CNVs, copy-number variants; het, heterozygosity; hemi, hemizygosity; homo, homozygosity; cohet, compound heterozygosity; AD, autosomal dominant; AR, autosomal recessive; XLD, X-linked dominant; XLR, X-linked recessive

Among the 82 subjects with genetically diagnosed NDD-NDCs, we identified 89 variants of which 37 were likely pathogenic and 52 pathogenic, based on the ACMG guidelines; 69 were SNVs in 48 genes, and 20 were CNVs. As illustrated in Fig. 3, missense variants were the most common variant type (34/89, 38.20%) in the genetically diagnosed cohort, while the remaining SNVs were truncation variants, including frameshift, nonsense, in-frame deletion, and splice variants. CNVs made up 22.47% of variants (20/89, 22.47%), and frameshift variants were the second most common SNVs (19/89, 21.35%), followed by nonsense variants (11/89, 12.36%). In-frame deletion and splice site variants were less common (3/89, 3.37% and 2/89, 2.25%, respectively) in the genetically diagnosed cohort. Of the 89 variants, 65 were *de novo* variants (DNVs), while the rest were non-DNVs. Of the 24 non-DNVs, 8 were inherited from one parent, while 16 and 2 were passed from both parents as compound heterozygous and homozygous variants, respectively. Meanwhile, the most common inheritance pattern in patients with SNV-mediated syndromes was autosomal dominant (37/62, 58.73%), followed by autosomal recessive (10/62, 16.13%), X-linked dominant (10/62, 16.13%), and X-linked recessive (5/62, 8.06%).

Among the 20 CNVs, 13 variants were likely pathogenic/pathogenic and involved one dosage-sensitive gene, and seven were likely pathogenic/pathogenic CNVs involving more than one dosage-sensitive gene. Specifically, 3 of the 7 pathogenic/pathogenic CNVs were known to cause syndromes, including 15q11-q13 microduplication syndrome (OMIM#608363), 2q31.1-q31.2 microdeletion syndrome (OMIM#142989), and Xp11.23 microduplication syndrome (OMIM#300801), while 3 were rare pathogenic CNV syndromes, each of which spanned dosage-sensitive genes related to NDD, including 12p13.33 microdeletion syndrome (*CACNA1C* involved), 6q25.3 microdeletion syndrome (*ERMARD* involved), and 19p13.2 microdeletion syndrome (*CACNA1A* involved). Finally, the last patient with a pathogenic CNV (patient 49) had a 16.07 Mb deletion at 9q31.1-q33.1 that spanned genes (*ZNF462* and *WHRN*) with unclear molecular function, but which were related to the subject’s phenotype (Supplementary File 4).

As shown in Supplementary File 8, no significant differences in GDD/ID severity were detected between cases with CNVs and those with SNVs (38/62 *vs* 15/20, *P* = 0.265), nor between those carrying DNVs and non-DNVs (41/65 *vs* 12/17, *P* = 0.564). Moreover, there was no significant difference in GDD/ID severity between patients with and without truncation variants (17/32 *vs* 21/30, *P* = 0.173).

### ASD risk variants categories and novel possible ASD-risk genes identification in patients genetically diagnosed with NDD-ASD

In the genetically diagnosed NDD-NDCs cohort, 64 patients had comorbid ASD, 48 of whom were carrying monogenetic variants in 37 genes, while 16 patients were carrying CNVs, including 15 dosage-sensitive genes. We divided the 37 genes with monogenetic variants in NDD-ASD cases into four classes based on the SFARI Gene database rating criteria. The majority of these genes (23/37, 62.16%) were clearly established ASD-related genes (Class 1), while six genes (6/37, 16.22%) were in Class 2, indicating that there was strong evidence for their association with ASD, and only one gene (*KAT6B*) was in Class 3, with suggestive evidence gene for association with ASD (Table 4). The remaining seven genes (*CUL4B*, *KCNH1*, *PLA2G6*, *SLC16A2*, *SSR4*, *UFC1*, and *WFS1*) were not present in the SFARI Gene dataset and were grouped into Class 4, which were considered candidate ASD-risk genes in our study.

**Table 4:** The categories of 37 genes of monogenetic NDD-ASD based on the ASD risk categories of SFARI Gene database

**Classes**	**Genes (case count)**
**Class 1:** category 1 (high-confidence) category S (syndromic ASD)	*ADNP* (1)*, ARHGEF9* (1)*, BRAF* (1)*, CDKL5* (1)*, CHD8* (1)*, CSNK2A1* (2)*, CTNNB1* (1)*, GRIN2B* (1)*, IQSEC2* (2)*, IRF2BPL* (1)*, KMT2A* (2)*, MECP2* (3)*, MTOR* (1)*, NSD1* (2)*, PPP2R5D* (3)*, PTEN* (1)*, SCN1A* (1)*, SCN2A* (1)*, SETBP1* (1)*, SETD5* (1)*, SRCAP* (2)*, STXBP1* (1)*, ZBTB20* (1)
**Class 2:** category 2 (strong evidence)	*KDM6A* (1)*, NR2F1* (1)*, RPS6KA3* (2)*, SPTBN1* (1)*, TPO* (1)*, TRRAP* (1)
**Class 3:** category 3 (suggestive evidence)	*KAT6B* (1)
**Class 4:** candidate ASD risk gene	*CUL4B* (1)*, KCNH1* (1)*, PLA2G6* (2)*, SLC16A2* (1)*, SSR4* (1)*, UFC1* (1)*, WFS1* (1)

*ASD* Autism spectrum disorder, *NDD-ASD* Neurodevemental delay and comorbid autism spectrum disorder

We also grouped the 15 dosage-sensitive genes from CNVs detected in patients with NDD-ASD into four groups, based on the available evidence regarding ASD-related genes and CNVs from the SFARI Gene dataset. As shown in Table 5, we found that four of the detected CNV syndromes were confirmed as related to ASD, among which two CNV syndromes (chromosome 15q11-q13 duplication and chromosome 15q11-q13 deletion) with two ASD-related genes (*GABRB3* and *UBE3A*, respectively) could be grouped in the “recorded CNVs with ASD-related genes” group. The other two CNV syndromes (chromosome 7q11.23 deletion and chromosome 1q21.1 deletion), including two uncertainly ASD-related genes (*ELN* and *GJA5*, respectively), were classified in the “recorded CNVs without ASD-related genes” group. Eleven CNV syndromes had not been frequently reported as associated with ASD; among which, seven CNV syndromes involving ASD-related genes, including *ARID2*, *CACNA1C*, *TBR1*, *ZNF462*, *CACNA1A*, *NFIX*, *PHIP*, and *STAG1*, were classified in the “unrecorded CNVs with ASD-related genes” group, and another four CNV syndromes with four uncertainly ASD-related genes (*BCL11B*, *FTSJ1*, *SHROOM4*, *IRF6*, and *WIZ*) were classified in the “unrecorded CNVs without ASD-related genes” group. According to the definitions for candidate ASD-risk genes in CNVs used in the current study, *ELN* and *GJA5* in the chromosome 7q11.23 and 1q21.1 deletions, respectively, were selected as candidate ASD-risk genes.

**Table 5:** The categories of 15 ASD-risk CNVs and included dosage-sensitive genes based on SFARI Gene database

**Categories**	**CNVs (case count)**
**Recorded CNVs with ASD-related genes:** Both the CNVs and their included dosage-sensitive genes have been proven to have close correlations with ASD phenotype	Chromosome 15q11-q13 duplication (*GABRB3* involved) (1), Chromosome 15q11-q13 deletion (*UBE3A* involved) (1)
**Recorded CNVs without ASD-related genes:** Only CNVs have been proven to have close correlations with ASD phenotype, and their included dosage-sensitive genes could be considered as candidate ASD risk genes in current research	Chromosome 7q11.23 deletion (*ELN* involved) (1), Chromosome 1q21.1 deletion (*GJA5* involved) (2)
**Unrecorded CNVs with ASD-related genes:** Only included dosage-sensitive genes have been proven to have close correlations with ASD phenotype	Chromosome 12p13.32 deletion (*ARID2* involved) (1), Chromosome 12p13.33 deletion (*CACNA1C* involved) (1), Chromosome 2q24.2 deletion (*TBR1* involved) (1), Chromosome 9q31.1-q33.1 deletion (*ZNF462* involved) (1), Chromosome 19p13.2 deletion (*CACNA1A/NFIX* involved) (1), Chromosome 6q14.1 deletion (*PHIP* involved) (1), Chromosome 3q22.3 deletion (*STAG1* involved) (1)
**Unrecorded CNVs without ASD-related genes:** None of CNVs and their included dosage-sensitive genes have been proven to have close correlations with ASD phenotype	Chromosome 14q32.13-q32.2 deletion (*BCL11B* involved) (1), Chromosome Xp11.23 duplication (F*TSJ1/SHROOM4* involved) (1), Chromosome 1q32.1-q32.3 deletion (*IRF6* involved) (1), Chromosome 19p13.2-p13.11 duplication (*WIZ* involved) (1)

*ASD* Autism spectrum disorder, *CNVs* Copy-number variants

**Fig. 4:**
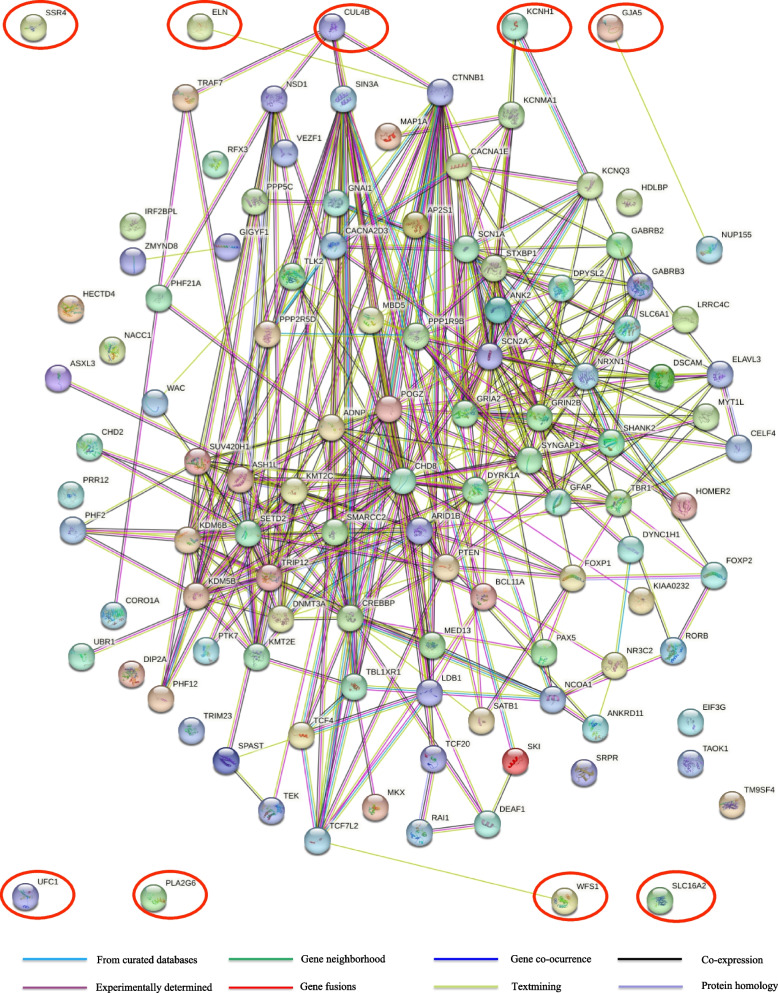
PPI network. The results of PPI analysis encompassing interactions among 9 candidate ASD-risk gene products (marked in red circles) identified in our work and 102 established ASD-related gene products obtained from the SFARI Gene database

To provide more evidence for identification of novel potential ASD-risk genes among Class 4 genes (*CUL4B*, *KCNH1*, *PLA2G6*, *SLC16A2*, *SSR4*, *UFC1*, and *WFS1*) and the two dosage-sensitive genes, *ELN* and *GJA5*, we conducted PPI network analysis using STRING. Nine candidate ASD-risk genes (seven genes related to SNVs and the two dosage-sensitive genes involved in CNVs) identified in the current study and 102 genes that are established as related to ASD (Supplementary File 2) were uploaded to the STRING online platform together and a PPI network generated (Fig. 4). No interaction with established ASD-associated genes was found for four genes (*SSR4*, *UFC1*, *PLA2G6*, and *SLC16A2*). For three genes, *ELN*, *GJA5* and *WFS1*, only text-mining-based interactions with established ASD-related genes (*CTNNB1*, *NUP155*, and *TCF7L2*, respectively) were detected, while two genes, *KCNH1* and *CUL4B*, had multiple connections, including experimentally determined, in curated databases, and co-expression, with two or four established ASD-related genes (*KCNMA1*/*KCNQ3* and *TRAF7*/*NSD1*/*CHD8*/*CTNNB1*, respectively), and could be considered novel possible ASD-risk genes. Moreover, among genes with no interactions with established ASD-related genes, we found four patients with *PLA2G6* variants who presented with an ASD phenotype in our cohort (two cases) and the cohort reported by Gregory et al. (two cases) [43], based on a comprehensive literature review of the OMIM database. Taken together, these results indicate that three genes (*KCNH1*, *CUL4B*, and *PLA2G6*) were identified as novel possible ASD-risk genes in our study.

## Discussion

Among the 163 children with unexplained NDD-NDCs who underwent trio-WES in this study, 82 obtained a genetic diagnosis that could explain their neurological manifestations, representing an overall diagnostic yield of 50.3%. This trio-WES diagnostic rate was similar to that reported previously in a trio-WES analysis of patients with NDD and additional associated neurodevelopmental disorders (53.5%) [44], and higher than those of previous WES studies of NDD patients with or without additional associated conditions (27% to 39%) [45–47]. A recent systematic meta-analysis to determine the diagnostic rate of WES, including 30 studies with 584 patients with unexplained NDD, showed that the diagnostic yield for isolated NDD was 31%, and that for NDD with additional associated conditions was 53% [6]. Another previous WES study of NDD cases, with additional clinical phenotypes in 31 of 33 individuals, reported a higher overall diagnostic rate (57%) [48]. All NDD cases in our study had at least one accompanying NDC (Table 1). Therefore, our present findings suggest that the presence of NDD in addition to NDCs enriches the diagnostic yield in the context of comprehensive trio-WES analysis, including both SNVs and CNVs. Moreover, the overall diagnostic yield of 50.3% generated by trio-WES in our cohort was almost double that of previous studies of CMA (15% to 20%) [49] and targeted NDD-panel sequencing (NDD-PS) (11% to 32%) [50] for patients with unexplained NDD, strengthening the conclusion of a recent evidence-based clinical practice guideline developed by ACMG board directors that, compared with CMA or NDD-PS test, WES has a higher diagnostic yield and can be more cost-effective; the guideline strongly recommends that WES be considered as a first- or second-tier test for patients with unexplained NDD in the early diagnostic evaluation stage [51].

The severity of NDD reported in genetically diagnosed cases of NDD-NDCs in this study ranged from mild to profound, and almost 65% of subjects (53/82) presented with severe-profound disability (Table 2); while, in the non-genetically diagnosed group, almost 79% of subjects (64/81) presented with mild-moderate disability (Table 3), indicating severe-profound NDD may potentially increase the likelihood of genetic diagnosis underlying unexplained NDD-DNCs conditions. This result is consistent with previous reports that NDD caused by genetic factors may be more severe than those resulting from non-genetic factors, as the latter were usually mild [12]. Approximately 40.2% of genetically diagnosed individuals (33/82) had abnormal head circumference; patients with severe-profound GDD/ID were more likely to have head circumference abnormality than those with mild-moderate GDD/ID (26/53 *vs* 7/29, *P* = 0.028) in the genetically diagnosed NDD-NDCs group (Table 2). Further, this positive phenotypic correlation was also present in the non-genetically diagnosed group (5/17 *vs* 6/64, *P* = 0.032) (Table 3), indicating that head circumference abnormality (mainly microcephaly) may be an important clinical characteristics underlying NDD in patients with severe-profound disability, regardless of whether it is caused by genetic or non-genetic factors. We also found that genetically diagnosed cases with a severe-profound disability were more likely to have comorbid ASD than those with mild-moderate disability (45/53 *vs* 19/29, *P* = 0.043) (Table 2), while in the non-genetically diagnosed group, there was no significant association between NDD severity and ASD comorbidity frequency (7/17 *vs* 28/64, *P* = 0.849) (Table 3), indicating genetically diagnosed patients with severe NDD were more likely to comorbid with ASD due to the shared genetic backgrounds [52]. Genetic NDD may share a common genetic etiology with other cognitive disorders, like ASD [12]. Thus, it is reasonable to speculate that a patient with a more severe NDD phenotype caused by genetic factors is more likely to have more pronounced language development delays and social communication disorders, which may more easily meet the diagnostic criteria for ASD.

Importantly, by comparing phenotypic differences between genetically diagnosed and non-genetically diagnosed NDD-NDCs patients with multivariate binary logistic regression analysis, we found that the most strongly associated independent phenotypic features in patients with positive trio-WES results were severe-profound NDD, multiple NDCs and accompanying ASD comorbidity or head circumference abnormality (Fig. 2). We speculate that severe NDD, a broad spectrum of NDCs, ASD and head circumference abnormality, may share genetic backgrounds, which are all strongly connected to overlapping genetic factors, potentially leading to a higher trio-WES diagnostic yield. There are over 1000 genes implicated in ASD susceptibility listed in SFARI Gene database; the major gene functional categories of ASD-risk genes are gene expression regulation, such as transcription regulation and chromatin modification, and neuronal communication, such as ion channel regulation and synaptic function [16]. Alterations in gene expression regulation and neuronal communication functions are also established as genetically related to severe NDD and multiple NDCs [53]. Further, during the process of craniofacial development, reciprocal signaling or neuronal communication between neural crest cells and the craniofacial ectoderm are essential for regulating craniofacial morphogenesis and patterning [54]. Alterations in these gene expression regulation signals and related neuronal communication lead to disruption between craniofacial ectoderm and neural crest, resulting in a wide range of craniofacial malformations, among which head circumference abnormality is prominent [55]. In our genetically diagnosed children, we observed that many of the detected genes with SNVs were involved in gene expression regulation function (39/62, 62.9%) and neuronal communication function (13/62, 21.0%) (Supplementary File 4), which may explain the high incidence of NDD with severe disability, multiple NDCs, or accompanying ASD and head circumference abnormality in cases with positive trio-WES results in our study.

Using the SFARI Gene database, we screened nine candidate ASD-risk genes which have seldom been reported as associated with ASD: seven candidate ASD-risk genes related to SNVs in Class 4 (*CUL4B*, *KCNH1*, *PLA2G6*, *SLC16A2*, *SSR4*, *UFC1*, and *WFS1*) (Table 4), and two candidate dosage-sensitive genes related to ASD-causing CNVs in the “recorded CNVs without ASD-risk genes” group (*ELN* and *GJA5*) (Table 5). Of the genes in Class 4, *CUL4B*, *KCNH1*, and *PLA2G6* have been reported to cause a phenotype of ASD or ASD-like behavior in several cases [43, 56, 57]. Additionally, *WFS1* is reported to be closely associated with multiple psychiatric illnesses, including severe depression, psychosis, obsessive-compulsive disorder, and suicidal behavior [58]. Moreover, although both the chromosome 7q11.23 and 1q21.1 deletion syndromes were included in the SFARI Gene database, there were no involved dosage-sensitive genes that could explain the cause of ASD in these CNV syndromes. By constructing a PPI network among the nine candidate ASD-risk genes and 102 established ASD-related genes using STRING (Fig. 4), we found that *ELN* and *GJA5* had tentative interactions (text-mining) with two genes implicated in ASD (*CTNNB1* and *NUP155*, respectively), indicating that CNVs involving in *ELN* and *GJA5* may partially contribute to the phenotype of ASD or ASD-like behavior via direct *ELN*-*CTNNB1* and *GJA5*-*NUP155* interactions. Further, the tentative interaction between *WFS1* and *TCF7L2* may also help to explain the psychiatric illness phenotype in patients with *WFS1* variants; however, further experiments are needed to elucidate their interactions. More importantly, we found that *CUL4B* and *KCNH1* had strong interactions, including experimentally determined, co-expression, and in curated databases, with multiple ASD genes, such as *TRAF7*, *NSD1*, *CHD8*, *CTNNB1*, *KCNMA1*, and *KCNQ3*, which are all confirmed to have crucial roles in causing autistic behavior phenotypes [41], providing evidence to support *CUL4B* and *KCNH1* as potential novel ASD-risk genes. Therefore, we propose that *CUL4B* and *KCNH1* warrant further exploration in the near future.

For genes (*SSR4*, *UFC1*, *PLA2G6*, and *SLC16A2*) that had no interaction with the established ASD genes, we would like to emphasize, *PLA2G6*, which maps to chromosome 22q13.1 and was first identified in Chinese hamster in 1997. *PLA2G6* encodes a cytosolic calcium-independent phospholipase A2 type IV protein with an important role in cell membrane homeostasis [59]. In our cohort, two subjects with NDD-ASD phenotypes (patients 77 and 82) were found to have compound heterozygous disease-causing variants of *PLA2G6*, and were diagnosed with NBIA2B. We did not identify any interaction between *PLA2G6* and established ASD genes by PPI network analysis; however, ASD phenotype or diminished social interaction was previously reported in two patients with missense variants of *PLA2G6* [43]. Further, mutations in *PLA2G6* are confirmed as associated with the pathogenesis of numerous neurodegenerative disorders, including Alzheimer’s and Parkinson’s diseases [60]. Moreover, functional studies have revealed that *PLA2G6* is critical for remodeling of membrane phospholipids, cell-signal transduction, and cell proliferation or apoptosis in dopaminergic (DA) neurons [61]. A recent study found that DA neurons in the midbrain dopamine system were commonly dysregulated in numerous patients with syndromic ASD [62] and contributed to autistic behavioral manifestations in syndromic ASD model mice [63]. Thus, based on these experimental findings and those previously reported NBIA2B cases with ASD phenotype, combined with the two cases detected in our cohort, we hypothesize that *PLA2G6* may also be a novel possible ASD-risk gene, and warrants further research attention.

Our study has some limitations. First, it was a single-center retrospective study, which will inevitably have been affected by selection, information, or confounding biases in collection and analysis of patient clinical phenotypes, and the results of this study may be influenced by these biases. Second, our work included of a group of different disorders with various rare neurogenetic diseases. This kind of study is clearly restricted by subject heterogeneity and limited numbers. Moreover, due to insufficient understanding of these rare NDD-NDCs and the lack of regular follow-up observations of such individuals, some additional clinical information may have been missed. Finally, although previously reported cases and bioinformatic analysis have provided some valuable insights into novel genes possibly related to ASD risk, more functional experiments are still needed to corroborate our findings, and will be the focus of our future work.

## Conclusion

In summary, our study included a relatively large cohort of patients with unexplained NDD and NDCs (163 cases) from a single-center, and is the first retrospective cohort study conducted in China where all included patients with NDD-NDCs patients were analyzed by trio-WES analysis with an overall diagnostic yield of 50.3%. The main strengths of our study can be summarized as follows: (1) By comparing the phenotypic difference between NDD-NDCs patients with positive and negative trio-WES results, we suggested that trio-WES testing is recommended when unexplained NDD-NDCs patients suffer from severe-profound NDD or multiple NDCs, particularly those with accompanying ASD and head circumference abnormality, because of the increased likelihood of making a genetic diagnosis in those patients using trio-WES. (2) Moreover, we also identified the novel possible ASD-risk genes (*CUL4B*, *KCNH1* and *PLA2G6*) underlying genetic NDD conditions. Patients with pathogenic variants in these genes should be aware of potential risks of developing ASD during their disease courses. These findings, based on trio-WES, may benefit affected children and their families, in terms of cost-effectiveness, family planning and diagnostic evaluation.

### Supplementary Information

Supplementary Material 1. 

Supplementary Material 2. 

Supplementary Material 3. 

Supplementary Material 4. 

Supplementary Material 5. 

Supplementary Material 6. 

Supplementary Material 7. 

Supplementary Material 8. 

## Data Availability

The raw data of this study are available from Supplementary File 3, 4 and 7. Further inquiries can be directed to the corresponding authors via email with reasonable requests.
